# The Vestibular Implant: Hearing Preservation during Intralabyrinthine Electrode Insertion—A Case Report

**DOI:** 10.3389/fneur.2017.00137

**Published:** 2017-04-10

**Authors:** Raymond van de Berg, Florence Lucieer, Nils Guinand, Joost van Tongeren, Erwin George, Jean-Philippe Guyot, Herman Kingma, Marc van Hoof, Yasin Temel, Jacobus van Overbeeke, Angelica Perez-Fornos, Robert Stokroos

**Affiliations:** ^1^Department of Otorhinolaryngology and Head and Neck Surgery, Maastricht University Medical Center, Maastricht, Netherlands; ^2^Faculty of Physics, Tomsk State University, Tomsk, Russia; ^3^Service of Otorhinolaryngology and Head and Neck Surgery, Department of Clinical Neurosciences, Geneva University Hospitals, Geneva, Switzerland; ^4^Department of Neurosurgery, Maastricht University Medical Center, Maastricht, Netherlands

**Keywords:** vestibular implant, vestibular prosthesis, neural prosthesis, bilateral vestibular areflexia, bilateral vestibulopathy, vestibulo-ocular reflex, hearing preservation, electrode design

## Abstract

**Objective:**

The vestibular implant seems feasible as a clinically useful device in the near future. However, hearing preservation during intralabyrinthine implantation remains a challenge. It should be preserved to be able to treat patients with bilateral vestibulopathy and (partially) intact hearing. This case study investigated the feasibility of hearing preservation during the acute phase after electrode insertion in the semicircular canals.

**Methods:**

A 40-year-old woman with normal hearing underwent a translabyrinthine approach for a vestibular schwannoma Koos Grade IV. Hearing was monitored using auditory brainstem response audiometry (ABR). ABR signals were recorded synchronously to video recordings of the surgery. Following the principles of soft surgery, a conventional dummy electrode was inserted in the lateral semicircular canal for several minutes and subsequently removed. The same procedure was then applied for the posterior canal. Finally, the labyrinthectomy was completed, and the schwannoma was removed.

**Results:**

Surgery was performed without complications. No leakage of endolymph and no significant reduction of ABR response were observed during insertion and after removal of the electrodes from the semicircular canals, indicting no damage to the peripheral auditory function. The ABR response significantly changed when the semicircular canals were completely opened during the labyrinthectomy. This was indicated by a change in the morphology and latency of peak V of the ABR signal.

**Conclusion:**

Electrode insertion in the semicircular canals is possible without acutely damaging the peripheral auditory function measured with ABR, as shown in this proof-of-principle clinical investigation.

## Introduction

Bilateral vestibulopathy is the disease resulting in a reduced or absent function of the vestibular organs, the vestibular nerves, or a combination of both (1). It can lead to blurred vision (oscillopsia) and impaired balance and spatial orientation (2, 3). A significant decrease in quality of life and considerable high physical and socio-economic impacts have been reported (4, 5). Unfortunately, prognosis is poor, and existing therapeutic options are limited and with low yield (6–8). Therefore, some research groups suggested a vestibular implant to replace the function of the vestibular organs (9–12).

This implant, in a concept analogous to the cochlear implant, captures motion and processes this information into an electrical stimulus that is delivered by electrodes to the vestibular nerves (13). However, many challenges are still to be met in the development of this implant (2, 14, 15). One of them is to surgically implant the electrodes in such a way that hearing loss can be prevented. This is necessary, because many eligible candidates with bilateral vestibulopathy have normal hearing or only a moderate hearing impairment (up to 49%) (16).

At this moment, two surgical strategies have been described: the intralabyrinthine approach (17) and the extralabyrinthine approach (18–20). With the intralabyrinthine approach, each semicircular canal is opened, and the electrodes are inserted up to the sensory epithelium of the ampullary nerves (17). With the extralabyrinthine approach, the labyrinth is not opened, and the electrodes are placed directly on the nerves. The nerves are reached by using the surgical approach to the posterior ampullary nerve (first described by Gacek) (20) and an approach developed to gain access to the lateral and superior ampullary nerves by removing the head of the malleus and incus and drilling superior to the prominence of the facial canal (18). Possible drawbacks of the extralabyrinthine approach are the certain degree of conductive hearing loss as a result of removing and reconstructing parts of the ossicular chain, possible damage to the facial nerve when drilling in the vicinity of the nerve, and not always being able to reach the ampullary nerves. Main advantages are a close proximity of the electrodes to the nerves and a low risk of inducing sensorineural hearing loss (17). The intralabyrinthine approach is used most often, probably because the extralabyrinthine approach is surgically more challenging with respect to reaching the site of stimulation. After all, the intralabyrinthine approach involves a “routine” mastoidectomy with bluelining of the semicircular canals, while the extralabyrinthine approach involves two more specific approaches. The main disadvantage of the intralabyrinthine approach is the fact that hearing can be compromised when the labyrinth is opened, and the electrodes are inserted in the semicircular canals (15). The Geneva–Maastricht group has only implanted patients who were deaf in the implanted ear because of this reason. Another group has implanted four Meniere’s patients with residual hearing, and all of them lost hearing following surgical implantation (21). However, this not necessarily implies that hearing will always be affected by electrode implantation in the semicircular canals. Research in rhesus monkeys has shown that hearing preservation is possible with the intralabyrinthine approach (22–24). Therefore, the main challenge of this approach is to optimize the surgical technique and to develop corresponding electrodes that can facilitate effective stimulation and at the same time preserve (residual) hearing in the majority of patients. The objective of this case study was to evaluate whether hearing can be preserved during the acute phase after electrode insertion in semicircular canals. The peripheral auditory function was monitored using auditory brainstem response audiometry (ABR) measurements, since this technique is highly sensitive in detecting auditory damage (25).

## Methods

### Patient

**Figure 1:**
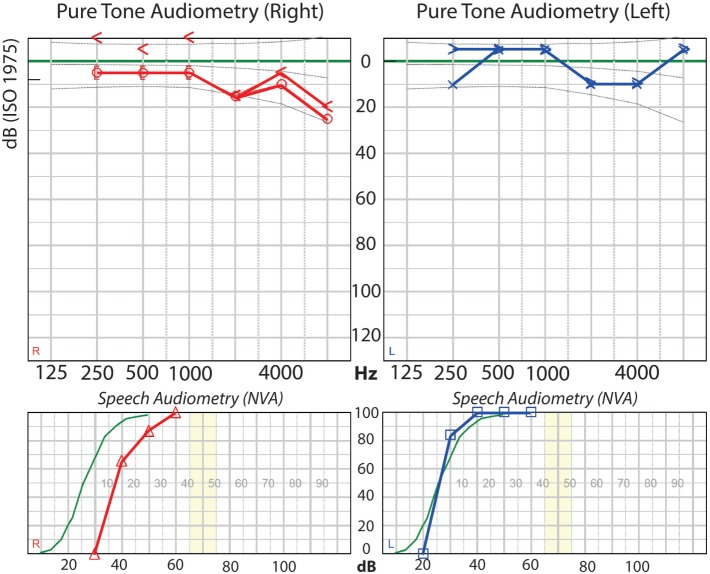
**Preoperative audiogram of the patient**.

A 40-year-old woman who had to undergo a planned partial resection followed by radiosurgery of a vestibular schwannoma Koos Grade IV on her right side was selected. She still had normal hearing, with a Pure Tone Average tone threshold (averaged over 1,000, 2,000, and 4,000 Hz) of 10 dB on the right side and 5 dB on the left side (Figure 1). Considering tumor size, and accordingly, the little chance of hearing preservation with any form of treatment, a translabyrinthine approach was chosen.

### Monitoring Peripheral Auditory Function and Video Recordings of the Surgical Procedure

**Figure 2:**
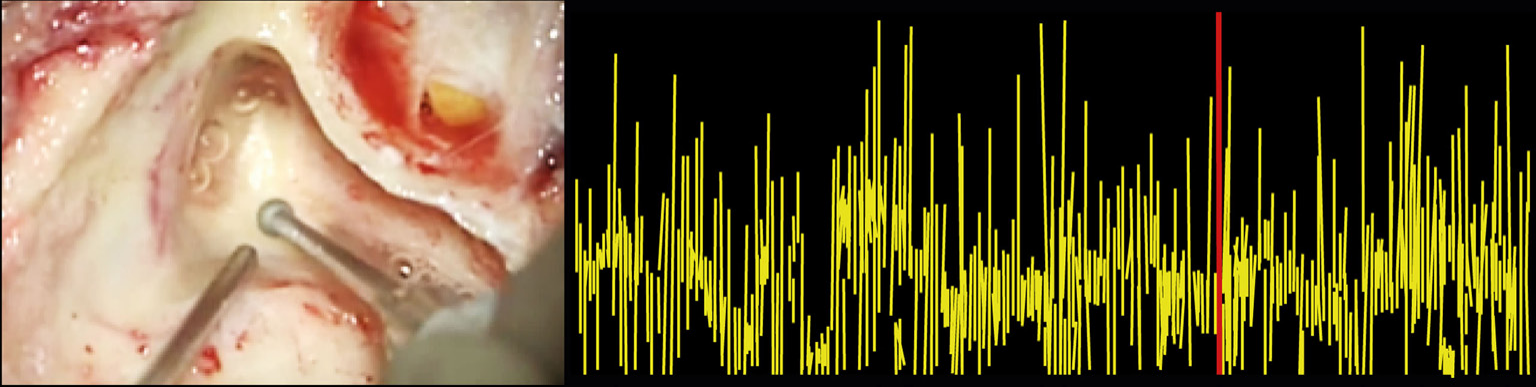
**Auditory brainstem response audiometry (ABR) artifacts during drilling**. The left panel shows the surgical drilling, and the right panel shows the synchronized ABR signal. It illustrates that surgical drilling induces ABR artifacts, making it impossible to draw any precise conclusions about the ABR signal during drilling.

Functionality of the cochleovestibular neuronal pathways was peroperatively monitored by ABR (25). An experienced audiological assistant continuously assessed ABR results. In order to be able to precisely measure the response, surgical drilling was paused during ABR measurements, since major artifacts occurred when drilling was performed (Figure 2). The ABR procedure was modified to be compliant with the intraoperative setting in two ways. First, the occurrence and stability of wave V as rated in consensus by three authors (Erwin George, Raymond van de Berg, and Joost van Tongeren) was deemed more important than a stereotypical morphology of the ABR signal, in a trade-off to surgical time. Wave V is thought to reflect the hearing level sufficiently for prediction purposes, which is why this was chosen instead of the other ABR waveforms (26). The presence of this peak could readily be determined after averaging approximately 500–700 measurements, whereafter the surgical procedure con-tinued. Second, the electrodes were placed just frontal of the ear lobes at frontal temporal-points FT9 and FT10, to prevent obstructing the surgical field. Ground and reference electrodes were placed at the forehead at frontal parietal-points FP1 and FP2. These are conventional electrode positions (27). ABRs were measured using a Viasys Health Care Synergy device (CareFusion, Palm Springs, CA, USA) and insert headphones, with click-type auditory stimulation at a rate of 11.7 pulses per second. The ipsilateral stimulation level was 80 dBnHL, with a contralateral broad-band masking noise at a level of 40 dBnHL. After surgery, pure tone audiometry was repeated.

Video recordings of the surgery and of the ABR signals were made by capturing the images of the surgical microscope and the images of the computer screen of the ABR equipment. The recordings of the surgery and the ABR signals were synchronized.

### Surgery and Study-Specific Procedures

**Figure 3:**
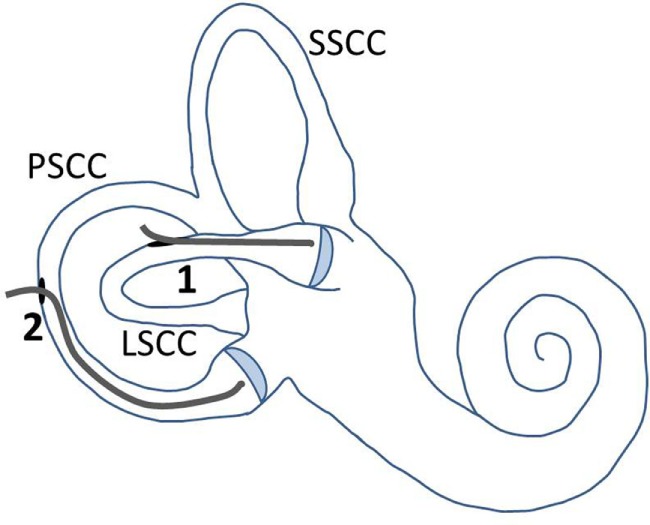
**Illustration of electrode placement and fenestrations in the semicircular canals**. The LSCC and PSCC were fenestrated at points away from their ampullae. The electrode was first inserted into the LSCC, up to the cupula (1). After removal of the electrode out of this canal, it was inserted into the PSCC, up to the cupula (2). Abbreviations: SSCC, superior semicircular canal; LSCC, lateral semicircular canal; PSCC, posterior semicircular canal.

Surgery involved a routine translabyrinthine approach. However, after the mastoidectomy and identifying the semicircular canals, the labyrinth was not immediately opened, and the additional study procedure started. This procedure involved the following steps. First, the lateral semicircular canal (LSCC) and posterior semicircular canal (PSCC) were bluelined, and the peripheral auditory function was monitored. Second, the LSCC was opened first, without disrupting the membranous labyrinth, and the peripheral auditory function was monitored again. Third, a conventional dummy electrode (made of silicone, with a diameter of 0.4 mm apical and 0.6 mm basal) was inserted in the LSCC approximately up to the cupula, and the peripheral auditory function was monitored. Following the principles of soft tissue surgery, the insertion was performed extra slowly to avoid strong hydraulic forces that could be transmitted throughout the inner ear. The electrode was inserted until a resistance was met, and from insertion depth it was deduced that the tip of the electrode was adjacent to the ampulla (Figure 3). This site was chosen since ampullary stimulation had previously resulted in reliable and reproducible electrical stimulation of the peripheral vestibular system (13, 17). Additionally, the electrode was lubricated with sodium chloride 0.9% before insertion, to diminish shear forces. No additional measures were taken to prevent any leakage from endolymph. Fourth, surgery was stopped for a couple of minutes after which the peripheral auditory function was monitored to investigate any “delayed” damage in the acute phase after insertion. Fifth, the dummy electrode was gently taken out of the semicircular canal, and the peripheral auditory function was monitored once more. The same steps, except for the fourth step, were then applied for the PSCC. Finally, the surgery for the vestibular schwannoma was continued. During this procedure, the peripheral auditory function was monitored after completely opening the three semicircular canals and after opening the vestibulum. The order of steps and their timing (commencing from the end of bluelining) are presented in Table 1.

**Table 1:** **Steps of study procedure**.

	Time
End of bluelining semicircular canals	0:00
Auditory brainstem response audiometry (ABR)	0:59
Lateral semicircular canal (LSCC) opened	5:00
ABR	5:53
LSCC electrode inserted	8:25
ABR	9:33
ABR	11:42
LSCC electrode taken out	12:19
ABR	14:13
Posterior semicircular canal (PSCC) opened	15:42
ABR	16:31
PSCC electrode inserted	18:29
ABR	19:25
PSCC electrode taken out	19:44
ABR	20:52
All 3 semicircular canals completely opened	24:20
ABR	25:19
Vestibulum opened	30:04
ABR	30:59
ABR	32:12
ABR	33:48
ABR	34:40

### Analysis of Data

The synchronized ABR video recordings of the surgery were analyzed in consensus by three authors (Erwin George, Raymond van de Berg, and Joost van Tongeren). Regarding surgery, special attention was paid to the observation of damage to the membranous labyrinth and leakage of endolymph.

## Results

### Surgery

All surgical steps of the study procedure and removal of the vestibular schwannoma were performed without complications. When opening the LSCC and PSCC, the membranous labyrinth was not disrupted at the point of fenestration. After insertion of the electrode and taking it out again, damage to the membranous labyrinth was observed in the lateral as well as the PSCC. However, no leakage of endolymph was observed, until the semicircular canals were opened completely as a part of the translabyrinthine surgery of the schwannoma.

### Peripheral Auditory Function

**Figure 4:**
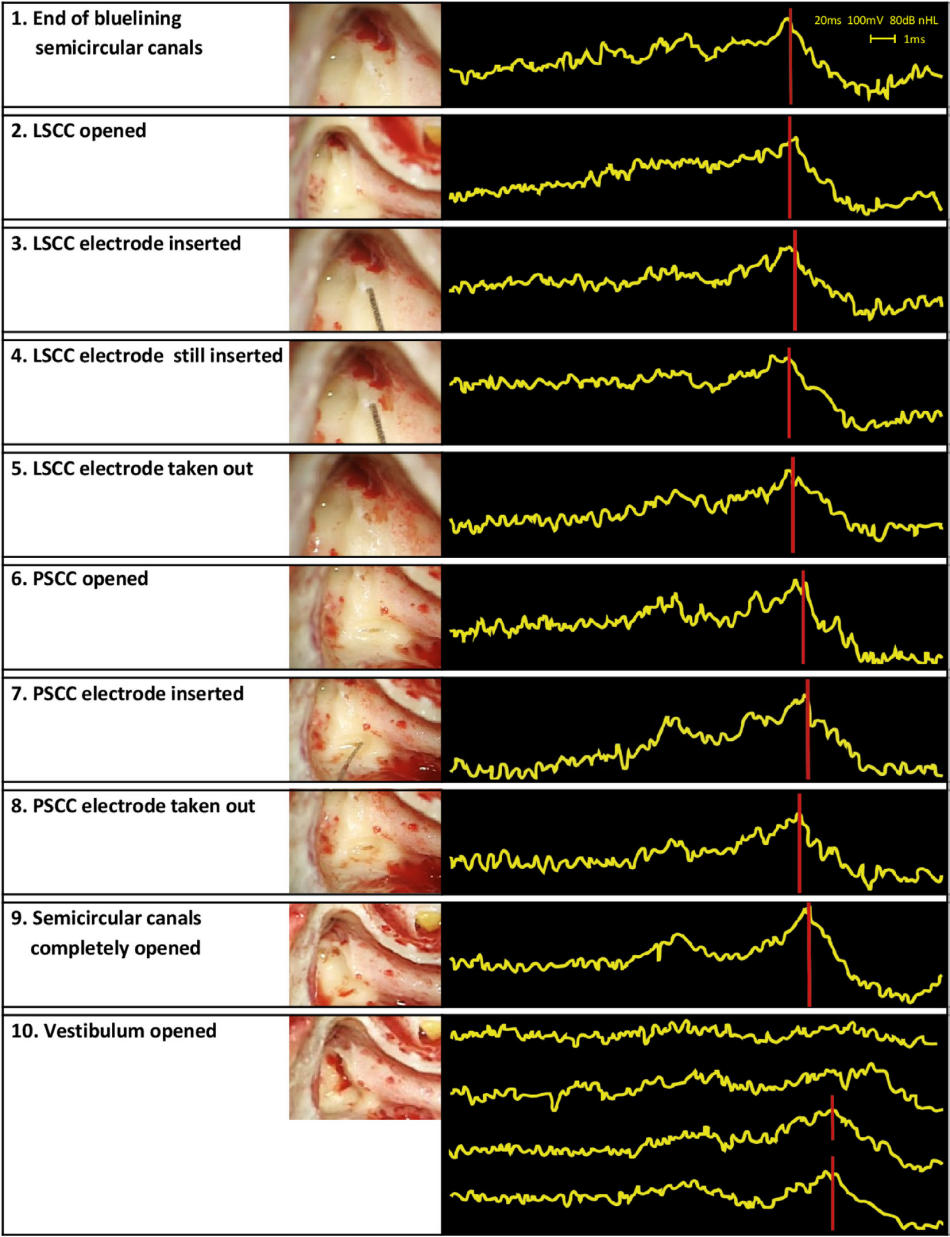
**Surgical steps and obtained auditory brainstem response audiometry (ABR) signals**. The left column presents the surgical steps, the right column the ABR measurements obtained during those steps. In the last row, four ABR signals are shown in the same box. These signals illustrate the last four ABR measurements of the study procedure. Their timing can be found in Table 1.

Auditory brainstem response audiometry results showed a stable morphology and a consistent peak V during the first 11 min after opening the LSCC. That is, the functional status of the cochleovestibular pathway appeared to be stable even after the LSCC was opened and after the electrode was inserted and removed. After opening the PSCC, peak V was still clearly present but it was delayed approximately 0.5 ms compared to baseline. ABR morphology, however, remained normal, and peak V remained present during opening of the PSCC and during insertion and removal of the electrode. Even after completely opening all three semicircular canals, ABR results appeared stable, with still a clearly visible peak V. However, after opening the vestibulum, ABR no longer showed any identifiable peaks, implying that functional hearing was clearly distorted. A largely delayed peak V seemed to return at a later moment, but morphology of the ABR signal and thus functional hearing still appeared to be compromised at this stage of surgery (Figure 4). The post-operative pure tone audiometry showed complete deafness of the operated ear.

## Discussion

The objective of this case study was to evaluate whether hearing can be preserved during the acute phase after electrode insertion in semicircular canals. It was shown that intralabyrinthine electrode insertion is possible without inducing significant damage to the peripheral auditory function measured by ABR, in the first half hour after opening the labyrinth and subsequent electrode insertion and removal. This is congruent with the experience in other intralabyrinthine surgeries such as semicircular canal plugging for intractable benign paroxysmal positional vertigo or superior canal dehiscence syndrome, in which hearing is often preserved (28, 29).

Major difference between electrodes in this study and “plugs” in previous studies is the fact that electrodes do not immediately close the whole semicircular canal and that they might penetrate the membranous labyrinth. This is important, since leakage of endolymph and perilymph is generally believed to induce loss of cochlear and vestibular function (28, 30). In this case, although the semicircular canals were not completely closed after insertion, no evident leakage of endolymph was observed. Next to this, no significant damage to the peripheral auditory function was observed, which is still in line with the hypothesis of endolymph leakage leading to hearing loss.

However, in another research group, previous vestibular implantations with electrodes designed to be selectively inserted in the perilymphatic space of the semicircular canals, resulted in hearing loss in all four Meniere’s patients following the implantations. Taken this into account, probably multiple factors could influence hearing during intralabyrinthine surgery. First, regarding the leakage of endolymph, patients with a hydrops (such as Meniere’s patients) or a gusher could be more susceptible to hearing loss (21, 31). When the membranous labyrinth is opened, the increased pressure in the endolymphatic system could probably be stronger than the surface tension of the perilymph and endolymph at the level of the fenestration. This could lead to excessive leakage of endolymph and perilymph and subsequent hearing loss. However, it has to be noted that no endolymph leakage was observed in the previously mentioned Meniere’s patients (personal communication Raymond van de Berg with James O. Phillips). Second, manipulating at a part of a semicircular canal furthest from the ampulla and vestibule is least likely to damage the other inner ear structures (28). Third, electrode insertion can induce hydraulic forces that are transmitted throughout the inner ear. These forces could violate the other inner ear structures (32). Fourth, a competent valve of Bast could preserve hearing, at least for some period of time (33). A combination of these factors might explain the initial only little timeshift of the ABR signal after completely opening the semicircular canals during this experiment and the apparent reoccurrence of peak V (with altered morphology) after opening the vestibulum.

### Future Investigations

Most of the abovementioned factors remain still partially speculative. Therefore, it should still be investigated which factors contribute to which extent to the occurrence of hearing loss during the acute phase of intralabyrinthine surgery. Until then, it could be advised to use “soft surgical” skills like preservation of the membranous labyrinth as long as possible, slow insertion of the electrodes, and avoiding suction directly on the perilymph or membranous labyrinth. Furthermore, it could be considered to open the labyrinth more far away from the ampulla and to not yet select patients with a suspicion of endolymphatic hydrops or gusher. In these cases, an extralabyrinthine approach might at this point be the safest procedure, since the labyrinth is not opened. Ideally, vestibular implantation surgery should be standardized. This would imply a safe surgical procedure and an optimal electrode design that complement each other, while both taking into account anatomical variabilities. At this moment, no optimal surgical procedure and electrode design are present yet (15, 17, 21, 24), but a standardized surgical technique with a complementary electrode design are currently being investigated (manuscript in preparation).

Regarding monitoring of hearing, the use of other (complementary) techniques could be considered like electrocochleography, or directly recorded cochlear nerve action potentials, in order to improve specificity. This, since ABR measurements are highly sensitive in detecting auditory damage, but specificity is poor (25).

Previous unsuccessful attempts for hearing preservation during vestibular implant (21) might have resulted in the impression that intralabyrinthine electrode insertion results in hearing loss. This case study shows that no damage of the peripheral auditory function was observed in the acute phase after electrode insertion. This at least raises the question whether hearing might be preserved in some cases after all. Therefore, after this proof of concept, the procedures of this study should be conducted in more patients to investigate reproducibility (34). However, it has to be noted that (at least in our centers) not many vestibular schwannoma patients with sufficient residual hearing are eligible for translabyrinthine surgery. The effort will be made by including more subjects to investigate the frequency of (partial) hearing preservation.

Finally, after a protocol for hearing preservation in the acute stage of intralabyrinthine insertion is established, the long-term effects should still be investigated. For example, delayed neo-osteogenesis, reactive fibrous tissue formation around the array, and delayed inner ear toxicity (blood, irrigation fluids, and device material) might all appear and could lead to delayed hearing loss (35).

### Limitations of the Study

Due to obvious ethical limitations, the time for the study procedure was limited. This resulted in two main limitations. First, not all semicircular canals were inserted with an electrode. At this moment, intralabyrinthine vestibular implants in humans consist of three electrode branches that are inserted in each semicircular canal (13, 21). Future studies will therefore need to have altered procedures (e.g., insert all electrodes at the same time, instead of sequentially) or more time scheduled for the procedures in their ethical approval. Second, the exact time point of complete hearing loss was not investigated. The presumably temporal reoccurrence of peak V, however, seems to indicate a dynamically changing hearing status during surgery. Factors contributing to this dynamic behavior in relation to the limitations of ABR should be addressed in future studies.

## Conclusion

Electrode insertion in the semicircular canals is possible without acutely damaging the peripheral auditory function as measured with ABR and shown in this proof-of-principle clinical investigation.

## Ethics Statement

The procedures in this investigation were in accordance with the legislation and ethical standards on human experimentation in the Netherlands and in accordance with the Declaration of Helsinki (amended version 2013). Approval was obtained from the ethical committee of Maastricht University Medical Center (NL54761.068.15/METC162006). All procedures were performed at Maastricht University Medical Center.

## Author Contributions

All authors contributed extensively to the work presented in this paper. JT, RS, and YT were the surgeons in this case. EG analyzed the ABR measurements. RB wrote the manuscript. FL created and edited the figures of the manuscript. FL, NG, JT, EG, J-PG, HK, MH, YT, JO, AP-F, and RS reviewed the manuscript and edited the manuscript.

## Conflict of Interest Statement

The authors declare that the research was conducted in the absence of any commercial or financial relationships that could be construed as a potential conflict of interest.
